# Proximate effects of temperature versus evolved intrinsic constraints for embryonic development times among temperate and tropical songbirds

**DOI:** 10.1038/s41598-017-00885-3

**Published:** 2017-04-18

**Authors:** Riccardo Ton, Thomas E. Martin

**Affiliations:** 1 Montana Cooperative Wildlife Research Unit, University of Montana, Missoula, MT 59812 USA; 2 U.S. Geological Survey, Montana Cooperative Wildlife Research Unit, University of Montana, Missoula, MT 59812 USA

## Abstract

The relative importance of intrinsic constraints imposed by evolved physiological trade-offs versus the proximate effects of temperature for interspecific variation in embryonic development time remains unclear. Understanding this distinction is important because slow development due to evolved trade-offs can yield phenotypic benefits, whereas slow development from low temperature can yield costs. We experimentally increased embryonic temperature in free-living tropical and north temperate songbird species to test these alternatives. Warmer temperatures consistently shortened development time without costs to embryo mass or metabolism. However, proximate effects of temperature played an increasingly stronger role than intrinsic constraints for development time among species with colder natural incubation temperatures. Long development times of tropical birds have been thought to primarily reflect evolved physiological trade-offs that facilitate their greater longevity. In contrast, our results indicate a much stronger role of temperature in embryonic development time than currently thought.

## Introduction

Embryonic development time varies greatly across species and latitudes often independently from body mass^1^. Classic theory posits that slower development reflects intrinsic constraints caused by evolution of physiological trade-offs that benefit phenotypic quality and longevity via enhanced tissue differentiation^2^, quality of immune responses^3^, and locomotor abilities^4^. However, slower embryonic development increases exposure to time-dependent mortality such as predation^5, 6^. Despite these important implications for fitness, the extent to which slower development reflects differences in evolved intrinsic constraints among species remains unclear.

**Figure 1:**
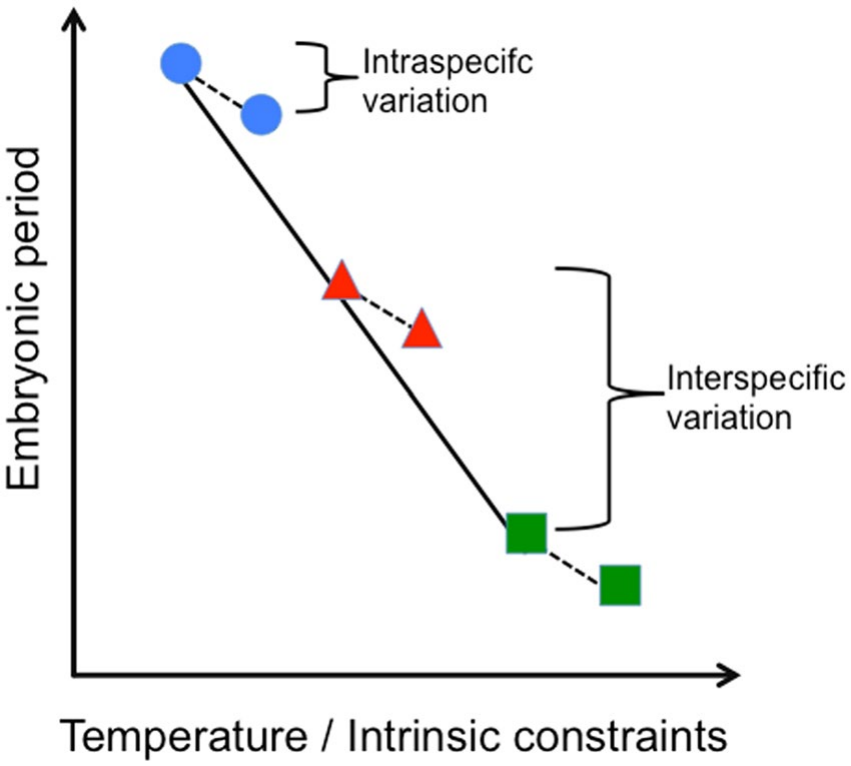
Conceptual graph representing the potential effect of temperature on embryonic development. Each symbol and color represents a different species. At the intraspecific level, temperature (dashed lines) can explain small differences in development period (small bracket). However it is unclear if temperature (solid lines) or physiological constraints are the main determinant of the larger interspecific differences in embryonic period (large bracket).

This uncertainty arises primarily because slower development can also occur in response to colder temperatures within species^7, 8^. Still, intraspecific variation in embryonic development time is small compared to variation among species (Fig. 1) and the role of temperature in influencing this larger interspecific variation remains poorly tested. Thus variation in embryonic development time may reflect traditional expectations of genetically based physiological programs yielding small variation in development time within species, and larger differences among species. Alternatively, selection may instead act on evolution of parental behaviors (e.g., nest/egg site choice, warming behaviors) that affect embryonic temperature^9–14^. As a result, the differences in intraspecific and interspecific variation in development time may reflect relatively consistent embryonic temperature within species, and larger differences among species (Fig. 1).

Understanding the relative roles of these two potential causes of interspecific variation in rate of embryonic development is critical because they yield opposing consequences for phenotypic quality and survival. Physiological trade-offs that extend development time can increase offspring quality^15–17^, whereas shorter embryonic periods can lead to smaller size at birth, higher metabolic rate and lower survival^18–20^. In contrast, cooler temperatures yielding longer development time generally create costs to offspring^21, 22^, while warmer conditions may yield beneficial effects^23^. Thus, consequences of interspecific variation in embryonic period can differ depending on the relative roles that physiological trade-offs versus temperature play in determining development time.

These two alternatives are strongly exemplified by songbird species living at different latitudes. Tropical songbirds commonly have longer embryonic periods that are correlated with higher adult survival compared with north temperate species^14, 24^. This correlation may reflect the classic expectation of physiological benefits from slower growth and development yielding higher adult survival and longer life^25^. Yet, the higher adult survival of tropical birds can also favor reduced parental warming effort and the resulting cooler embryo temperatures may cause their longer embryonic periods^26^. Thus, this last possibility reverses the direction of causality between embryonic development time and adult survival and highlights the importance of understanding the basis of interspecific variation in embryonic development. Untangling these alternatives requires an experimental approach that has until now not been attempted in a temperate-tropical context.

Here we conduct controlled heating and egg-swap experiments in tropical and north-temperate species that exhibited broad differences in embryonic development times and incubation temperatures. We also compared differences in egg mass loss, metabolic rates, and hatching success between treatment and control nests to test for potential costs to embryos associated with faster development at warmer temperatures. This set of experiments allows us to untangle the role of incubation temperature and physiological constraints on embryonic development time and their possible consequences.

## Results

### Heating Experiment

Our heating experiments at 42 nests successfully increased average incubation temperatures (mean ± SE = 1.32 ± 0.13 °C; see actual temperatures measured for control and treatment nests by species in Supplementary Table S1) and did not affect hatching success. Hatching success is typically about 90% in natural nests^27^. In our study, 93 ± 0.7% of eggs hatched, with no differences between treatment and control clutches (*F*_1,342_ = 0.213, *P* = 0.69).

**Figure 2:**
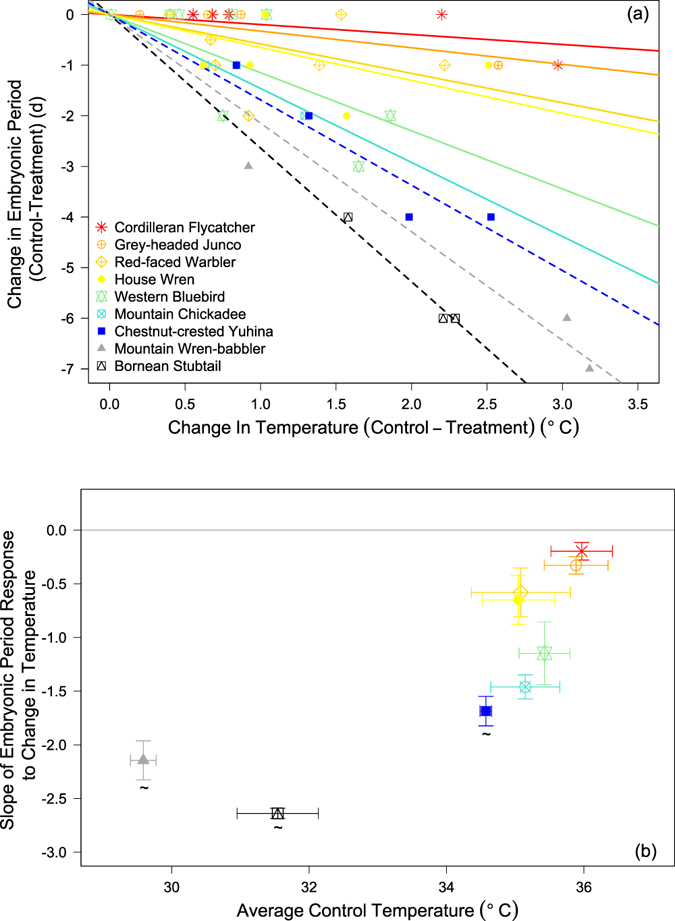
(**a**) Correlation between measured differences in egg temperature, and incubation period differences between treatment and control among 42 paired nests belonging to nine species at two latitudes. Each point represents a nest pair and each symbol and color a different species. Individual regression lines provide the intraspecific response of embryonic period to experimental heating and warmer colors are associated to warmer natural incubation temperature. Dashed lines denote tropical species. Names in figure legend are reported in order of ascending slope. (**b**) Correlation between average (±1 SE) differences in control incubation temperature and change of embryonic period with treatment temperature (slope ±1 SE) for nine songbird species at two latitudes. Tropical species are denoted as ~. The gray horizontal line intercepting zero represents the physiological threshold for development where further temperature increases produces no changes in embryonic period.

Experimentally increased temperature was generally associated with a decrease in embryonic period among all nine study species (Fig. 2a). However, responses of individual species to our warming experiment varied substantially. Cordilleran flycatcher (*Empidonax occidentalis*) showed almost no reduction in embryonic period (mean ± SE = −0.2 ± 0.08 d), whereas Mountain wren-babbler (*Napothera crassa*) showed impressive shortening in development time (mean ± SE = 5.33 ± 1.2 d) with increased temperature (Fig. 2a). Slopes were steeper (i.e., stronger responses to temperature changes) in species that normally (i.e., controls) kept their eggs cooler (Fig. 2b, *r* ^*2*^ = 0.69, *P* = 0.005). Tropical species commonly have colder egg temperatures because of reduced time spent warming eggs by parents in the tropics^14, 26^. Consequently, embryonic periods of our tropical species with their colder normal egg temperatures exhibited stronger responses (steeper slopes) to heating compared with our north temperate species with warmer normal egg temperatures (*F*_1,7_ = 17.38, *P* = 0.004).

### Egg Swap Experiment

Bornean stubtail (*Urosphena whiteadi*) eggs placed in Chestnut-crested yuhina nests experienced higher average temperatures (mean ± SE = 4.06 ± 0.39 °C) and showed a 25% shortening in development time (mean ± SE = 6 ± 0.4 d) compared to controls. This reduced the average difference in development time between the two species from ten (mean ± SE = 10 ± 0.4 d) to four (mean ± SE = 4 ± 0.4 d) days. Therefore temperature alone accounted for 60 ± 5.8% of the difference in average embryonic period between natal and host species, while the remaining 40 ± 5.8% can be attributed to intrinsic constraints or other unmeasured variables. Physiological trade-offs also acted on the other end of the temperature gradient limiting the extent to which development can be delayed. Eggs of Chestnut-crested yuhina (*Yuhina everetti*) that were transferred to the colder temperature conditions of stubtail nests extended their embryonic period by two days on average (mean ± SE = 2 ± 0.4 d). A substantial difference from the host nest remained (mean ± SE = 8 ± 0.4 d) suggesting the presence of selection favoring fast growth independently from the effect of temperature. Thus, physiological trade-offs acted asymmetrically in these two species, with seemingly less intrinsic constraints in the slow (cold) than fast (warm) species.

### Consequences of increased temperature for egg mass and metabolism

**Figure 3:**
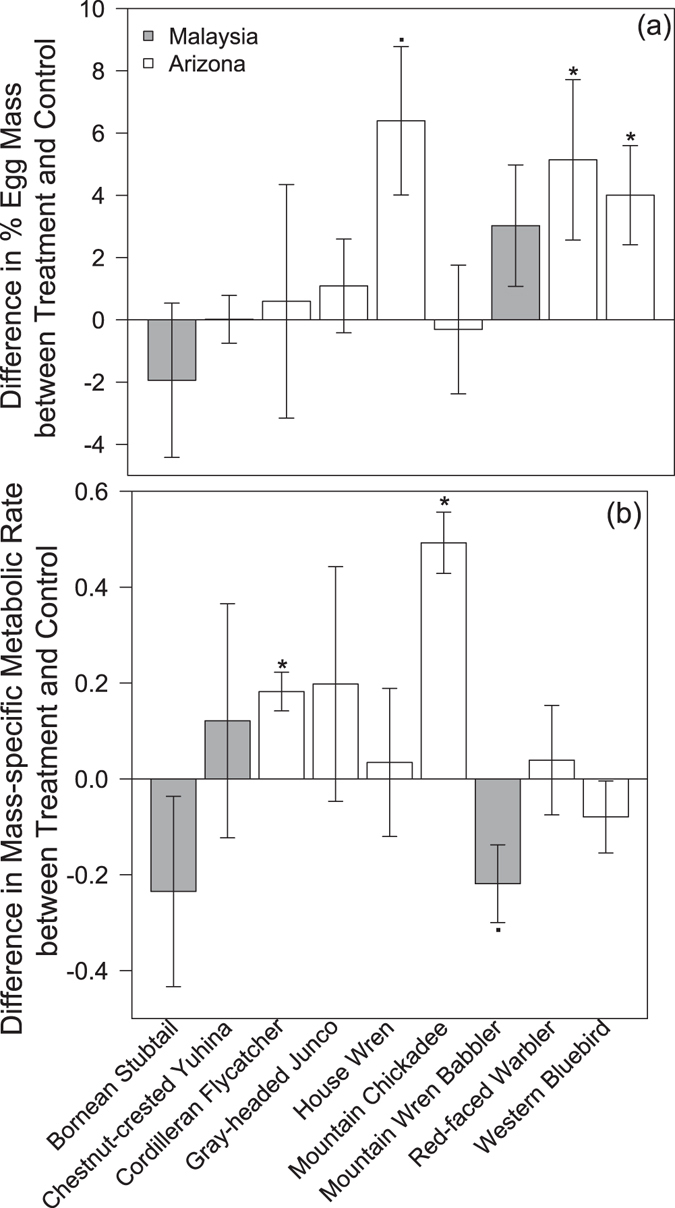
Mean differences between treatment and control (±1 SE) in (**a**) % egg mass, and (**b**) mass-specific metabolic rate (mL O_2_ h^−1^) for nine bird species exposed to increased incubation temperature in a tropical (Malaysia) and north temperate (Arizona) site. Significant (p < 0.05) and marginally significant (p < 0.1) effects within species are denoted respectively as * and ●.

Egg mass naturally decreases over the embryonic period due to water loss associated with metabolic processes underlying development. Average reduction in egg mass in our study was 14.6% ± 0.41 for all samples (*F*_1,90_ = 2.618, *P* = 0.014) but interspecific differences were substantial, ranging between 10–22%. Embryos of seven species lost less mass when exposed to heating, three of which were significant (3a). The remaining two species tended to lose more mass when incubated at warmer temperatures but the effect was not significant (Fig. 3a; Supplementary Table S2). Overall, warmer temperatures during development resulted in heavier embryonic mass prior to hatching across the nine species in our experiment (Fig. 3a; *F*_1,90_ = 9.91, *P* = 0.003).

Heated embryos had higher mass-specific metabolic rates than control embryos in six species, two of which showed a significant effect of treatment. Mass-specific metabolic rate was lower in three species and one of those showed a marginally significant effect of the heating experiment. This resulted in an overall lack of significant effect of our treatment on metabolic rates (Fig. 3b; *F*_1,90_ = 0.813, *P* = 0.372).

## Discussion

Differences among species in embryonic development and growth rates have long been viewed as a strong function of genetic programing and physiological processes that affect phenotypic quality and longevity^15, 25^. Of course, proximate factors (e.g., food, temperature) can influence development rates within species^28^, but such proximate effects have been considered a minor modifier of the variation among species. Yet, even species within the same order can differ dramatically (up to 7 °C) in the temperatures to which their embryos are exposed (Fig. 2b), because of differences in parental warming behavior^14, 26^. Such interspecific differences in embryonic temperatures are not restricted to birds^1^ and can vary among diverse taxa as a function of parental choice of oviposition and nest sites, basking, and brooding behaviors^9, 12, 13^. The substantial reduction in embryonic period in response to experimental heating (Fig. 2a) clearly demonstrates that such interspecific variation in temperature can strongly influence length of the embryonic period. Indeed, our average temperature increase of about 1.3 °C was much smaller than the differences in embryonic temperature that existed among species. Moreover, the change in embryonic period by as much as 7 days with warming (Fig. 2a) clearly shows that temperature can explain a substantial portion of the interspecific variation in embryonic development time. Our results suggest that the proximate effect of temperature can play a much stronger role in the differences in embryonic development time among species than previously thought, but temperature effects are greater among species with colder natural incubation temperatures (Fig. 2).

Ultimately, physiology appears to limit how short development time can be. The shortest period of embryonic development in birds is 10–11 days^29^, and this is typically seen when average incubation temperature is close to 37 °C^26^. Thus, embryos experiencing natural incubation temperatures near 37 °C may have evolved development periods close to their physiological limits. Species near their physiological maxima at the warm end of the temperature spectrum reduced their embryonic periods very little during warming experiments (Fig. 2). Rather, heating shortened embryonic period much more in tropical species with colder incubation temperatures and longer development times (Fig. 2). This strong role of temperature for the long development times of tropical birds is particularly interesting because tropical birds have been viewed as a good example of the benefits of long development times for longevity due to physiological trade-offs^15^. Yet, such arguments were predicated on the idea that genetically based physiological programs caused long development times by allocating relatively more intrinsic resources to physiological systems that enhanced longevity^2–4, 15^. Our results showing that long development times, as common in tropical birds^14, 26^, was a strong function of temperature rather than physiological programs raises questions about such perspectives.

Faster development may lead to reduced phenotypic quality like smaller mass at hatching in lizards^30^, and faster metabolic rates in domestic chickens^18^, both of which decrease survival^31^. Yet, we found that eggs developing faster at warmer temperatures were overall heavier prior to hatch date compared to control eggs among the nine species studied here (Fig. 3a). This result fits as the converse of egg cooling studies, which found cooling caused smaller and lower quality offspring in various taxa^32, 33^. This effect may be explained by warmer temperature favoring higher efficiency in cell differentiation and proliferation, whereas lower temperatures diverting resources to respiration and self-maintenance. Intriguingly, our reported effects of temperature on egg mass support the hypothesis that parents in the tropics lay larger eggs to provision their embryos with extra resources that compensate for the maintenance costs of low average incubation temperatures^34^. Also, we did not detect an overall change in mass-specific embryonic metabolic rate due to heating (Fig. 3b). The possibility remains that higher incubation temperatures underlying faster development may produce other costs unmeasured here with detrimental consequences for adult longevity, but we did not detect costs related to mass and metabolism.

Long development times may impose a cost on young when caused by low incubation temperatures in two ways. First, long development from cold temperatures requires increased use of internal resources by the embryo during development and yields smaller and lower quality offspring^32, 33^. Second, slow development at lower temperatures extends the time of exposure to sources of mortality experienced during the vulnerable embryo stage. For example, Bornean stubtail eggs have a 24-day incubation period and are exposed to a daily predation probability of 0.045^14^. Yet, our experiments show that this species has the potential to shorten embryonic development by at least six days, which translates into a 24% reduction in predation risk. Why then do songbirds not increase incubation effort to keep eggs warmer and shorten the incubation period so that their offspring benefit from reduced predation risk?

A possible answer is that costs of greater incubation effort accrue to parents rather than to offspring. In long-lived species selection may favor reduced energy expenditure by parents so that they enhance their own probability to breed in the future^10, 26^. Tropical birds commonly spend much less time keeping eggs warm causing colder average egg temperatures than north temperate relatives^14, 26^. Nevertheless, the egg swap experiment demonstrated that embryonic development time includes intrinsic constraints that may reflect beneficial physiological trade-offs. Thus, selection may favor increased longevity via two mechanisms that both yield longer development times: by acting on physiological trade-offs to improve phenotypic quality, and by reducing parental effort and resulting extrinsic embryonic temperature.

The effects that developmental temperature has on phenotypic variation are especially important in light of global climate change^35^. Our data show that “cold” tropical embryos shorten development time more than “warm” north temperate species for an equivalent increase in temperature. This suggests that small increases in temperature due to global warming predicted at low latitudes^36^ may benefit tropical embryos by shortening their incubation period and reducing exposure to predation with potentially minor phenotypic costs to the offspring. Conversely, increased temperatures in north temperate zones may yield smaller effects for development because species are closer to their physiological maxima (Fig. 2), causing tropical species to potentially be bigger ‘winners’ than temperate species from global warming^37^ with respect to development time and offspring predation risk. At the same time, parents of temperate species may be able to spend less time on the nest under warmer conditions and obtain survival benefits from increased self-maintenance. Thus, fitness costs of global warming between latitudes^38, 39^ are unclear in songbirds and deserve further research attention.

Our study shows that extrinsic temperature plays an increasingly stronger role than intrinsic constraints on interspecific variation in embryonic period among species with colder incubation temperatures. The response to warmer temperatures varied as a function of the thermal conditions normally experienced during development, leading to questions about possible latitudinal differences in the effect of global warming on ectothermic embryos. Additionally we found benefits rather than costs associated with shorter embryonic periods. These results will require revision of views on the relative importance of proximate effects versus intrinsic constraints for interspecific variation in embryonic development time, and the potential consequences for phenotypic quality and longevity.

## Methods

### Study Areas and Species

We focused on six songbird species between May and July 2011–2014 in a north temperate mixed forest at 2000–2350 m elevation in Arizona, USA (33°N). This work was conducted under the Arizona Game and Fish Department permits SP557502 and SP614321, and the U.S. Fish and Wildlife Service permit #MB791101-0. We studied three additional species between February and May 2012–2014 in a tropical forest at 1450–1750 m elevation in Sabah, Malaysia (6° N) (Supplementary Table S3). Work in Malaysia was authorized by the State of Sabah through Sabah Parks and the Sabah Biodiversity Council permits (Licence ref.no. JKM/MBS.1000-2/2 JLD.4 (40)). The nine species were chosen to represent a strong gradient in embryonic development time. All experiments and measurements were conducted in accordance with relevant guidelines and regulations under the auspices of the University of Montana IACUC protocol #059-10TMMCWRU. We conducted.

### Experimental Increase in Incubation Temperature

We increased incubation temperature at 42 treatment nests each paired with a control nest exposed to the same level of manipulation but experiencing natural incubation temperatures. Treatment and control nests were spatially and temporally matched in order to minimize differences in weather, seasonality, habitat and elevation. We also matched nests with the same clutch size because the number of eggs can influence embryonic development rates^40^. The experiment lasted for the full length of the embryonic period starting from the last egg laid and ending with the first egg hatching. One heating device (Kapton Heaters model #KHLV-105) was installed around the nest cup and powered by a 12 V car battery that we replaced every second day. Heat output from the device was regulated by a thermostat connected to a probe placed in the bottom of the nest (Pressure Tek, model# 3943) set at 37.5 °C (Supplementary Fig. S1). This value is considered around the optimum range for embryonic development^41^. Control nests were treated the same, except that the heating device was wired to a cardboard box to simulate the battery.

**Figure 4:**
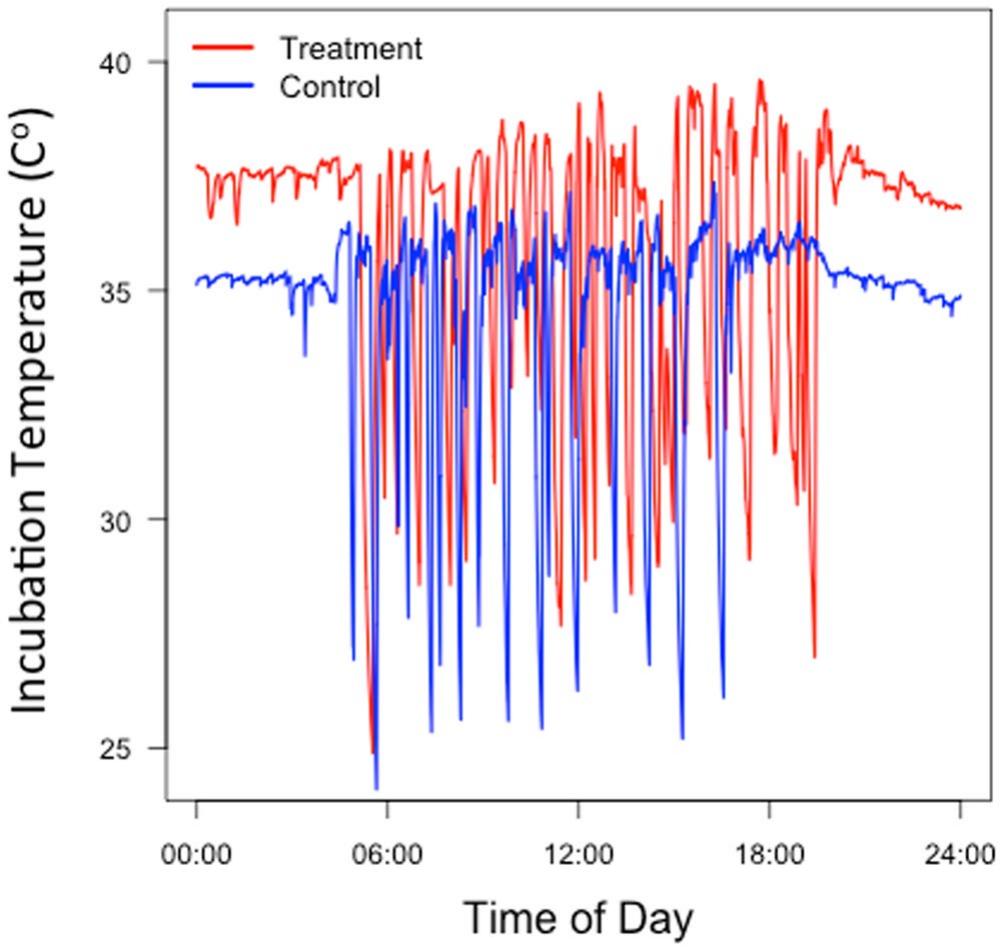
Thermal conditions experienced during 24 hours of incubation by a control and a treatment clutch of Red-faced warblers (*Cardellina rubifrons*) of the same age. Measurements were recorded on the same date and nesting habitat; temperature oscillations reflect parental incubation behavior, with downward spikes from parents leaving the nest to forage.

The overall effect of the heaters was to raise the temperature of the nest during periods when the parents were absent or when incubation temperatures were sub-optimal. Thus average 24-hr egg temperature was increased while maintaining normal incubation rhythms (Fig. 4) and avoiding heat stress to the embryos^42^. Nests were normally checked every 48 hours, but our monitoring effort increased up to four times daily as hatch dates approached. Embryonic period length was calculated as the number of days between the last egg laid and the first egg to hatch. To minimize loss of nests to predation, treatment and control nests of open-cup-nesting species were caged with iron mesh that allowed normal movements of parents but prevented most mammal and bird predators from accessing the nest.

### Egg Swap Experiment

At our tropical site during the 2014 season, we performed a second experiment that replicated methodology detailed in ref. 26. The goal was to test the relative contribution of temperature and physiological trade-offs for differences in embryonic period between two tropical species. Chestnut-crested yuhina (*Yuhina everetti*) and Bornean stubtail (*Urosphena whitheadi*) have comparable egg mass but embryos of the former experience temperatures 5° C warmer on average than the latter during development because of differences in parental incubation effort^43^. If development is caused by physiological trade-offs alone, swapped eggs should hatch at the same time as un-swapped controls of the same species. In contrast, if temperature is the sole cause of developmental period length, swapped eggs should show the same embryonic periods as their host nest species. Thus, by examining the relative change in developmental time in swapped eggs, we can partition the relative importance of intrinsic constraints versus temperature. Eggs were transferred in the morning during the laying stage (i.e., prior to start of incubation) between nests of the same stage. Neither of these two species starts incubating before all eggs are laid, so embryos were undeveloped at the time of swapping. Nest predation is reasonably high^14^, and seven pairs of experimental nests were lost despite protection provided by the cage. Yet we were successful in hatching swapped eggs between four pairs of nests.

### Temperature Measurements

We quantified temperature in all nests and in both experiments (Fig. 4) by placing a thermistor in the center of an artificial egg positioned in the middle of the nest and connected to a HOBO Stowaway XTI datalogger (Onset Computer Corporation, Bourne, Massachusetts, USA; Supplementary Fig. S1). The dummy egg recorded temperature every 12 seconds for three days halfway through the natural incubation period and was then removed. We chose to record temperature at this stage for three reasons. First, intra and interspecific variation in the amount of time spent brooding is higher during the early stages of incubation^44^. Second, incubating parents tend to be more sensitive to disturbance such as adding an extra egg to the clutch in early than middle incubation (personal observation). Third, we needed to take measurements before possible early hatch of treatment nests. Fake eggs were made of plaster of paris and were formed to mimic the size, shape and color of the host species. We limited our measurements of temperature differences to three days because larger clutches can increase the energetic costs of incubation to parents^45^.

### Egg Mass and Metabolic Measurements

We marked and weighed all eggs in our heating and swap experiment using an ACCULAB portable electronic scale (precision 0.001 g; Edgewood, NY, USA). A first weight was taken the day of clutch completion followed by a second measurement two days before the expected hatch date. We were able to forecast pretty accurately hatch date for all nests via “candling”. This practice consists in shining through the eggshell a powerful light source to monitor the extent of embryonic development. When approaching the last stages of incubation we were recording egg mass daily. We kept in our analysis only the data point taken two days before actual hatch date while discarding the others. These measurements allowed us to quantify the mass lost by each clutch between the stages of early and late incubation.

We also measured embryo metabolic rate in eggs as oxygen consumption rate [*V*O_2_ (mL h^−1^)] using a FoxBox field gas analyzer (Sable System, Las Vegas, NV, USA) for one egg only in order to ensure independence among samples. Metabolic measurements were executed on eggs from both our experiments and followed the protocol detailed in ref. 43. Eggs were removed from the nest at 79.9 ± 0.72% (mean ± SE) of their development and were replaced with fakes. During metabolic measurements, the eggs rested in a 60 mL syringe, connected to an open-flow system flushed with atmospheric air at a rate of 25 ml/min. The air was scrubbed of CO_2_ and water vapor using magnesium perchlorate, soda lime and drierite. To precisely control experimental temperature, the chamber was submerged in a water bath and held at 37.5 °C.

Oxygen consumption rate was measured continuously every 0.5 s, and *V*O_2_ (mL h^−1^) was calculated as the difference in O_2_ concentration between the air input and output flowing through the chamber during the most stable three minutes of measurements. We used the formula *V*_*O2*_ = FR_i_(F_i_o_2_ − F_e_o_2_)/(1 − F_e_o_2_) in ExpeData (ver. 1.3.2) software from Sable Systems. Where FR_i_ is the incurrent mass flow rate scrubbed from water vapor and CO_2_, F_i_o_2_ is the incurrent fractional concentration of oxygen, and F_e_o_2_ is the excurrent fractional concentration of oxygen^46^. Metabolic measurements lasted between 60 and 90 minutes, with larger eggs taking longer. After completion each egg was returned unharmed to the nest of origin.

### Statistical Analyses

#### Experimental increase in incubation temperature

We tested the effect that differences in incubation temperatures between treatment and control nests have on embryonic period using a linear mixed model with species as a random effect nested within site and year. We forced all intercepts through zero^47^ because no difference in temperature should yield no difference in embryonic period (Supplementary Table S4).

To test for evolved differences among species in the responses of developmental time to temperature, we extracted the coefficients (slopes) from the previous model. We used these slopes as the dependent variable and average temperature in control nests as the independent variable in a linear model that took into consideration phylogenetic history (package “caper”). To produce our phylogeny we built a majority-rule consensus tree with program Mesquite using 1,000 trees sampled from BirdTree.org^48^ (Supplementary Fig. S2).

#### Egg swap experiment

We quantified the relative contribution of temperature versus intrinsic constraints for determining differences in embryonic period between species in our swap experiment. We first subtracted the length of embryonic period (days) of the egg remaining in the natural nest from the length of embryonic period of the egg transferred to the host nest. We then divided this change in embryonic period by the observed difference in embryonic periods of the host versus natal nest x 100. We attributed this percentage change in embryonic periods between species to temperature and the remaining portion to physiological trade-offs and other unmeasured effects.

#### Egg mass and metabolic measurements

We tested for effects of heating on egg mass loss by fitting a linear mixed model with percent egg mass loss difference between treatment and control as the dependent variable, a categorical variable with two levels (treatment and control) as independent variable and species as a random effect nested within site and year. Using the same statistical approach we tested for differences in mass specific metabolic rate and hatching success between treatment and control nests. We also conducted separate ANOVA tests for each species to evaluate whether differences in egg mass loss and mass-specific metabolic rate were significantly different from zero (Supplementary Table S2). All analyses were performed using the R package version 3.1.2^49^.

## Electronic supplementary material

Supplementary material
