# Supplementary material for: Proximate effects of temperature versus evolved intrinsic constraints for embryonic development times among temperate and tropical songbirds
Source: Sci Rep. 2017 Apr 18;7:895. doi: 10.1038/s41598-017-00885-3 (PMC5429855; doi:10.1038/s41598-017-00885-3)

**Supplementary material for:**

# Proximate effects of temperature versus evolved intrinsic constraints for embryonic development times among temperate and tropical songbirds.

Riccardo Ton and Thomas E. Martin

Table S1. Average incubation temperatures are shown for treatment and control nests of each species as measured by our artificial egg.

| **Site** | **Common Name** | **Scientific Name** | **Control Temperature *C*** | **Treatment Temperature *C*** |
|---|---|---|---|---|
| Malaysia | Bornean Stubtail | *Urosphena whiteadi* | 31.54 | 33.57 |
| Malaysia | Mountain Wren-babbler | *Napothera crassa* | 29.58 | 31.96 |
| Malaysia | Chestnut-crested Yuhina | *Yuhina everetti* | 34.57 | 36.23 |
| Arizona | Cordilleran Flycatcher | *Empidonax occidentalis* | 35.96 | 37.19 |
| Arizona | Grey-headed Junco | *Junco hyemalis* | 35.88 | 36.97 |
| Arizona | House Wren | *Troglodytes aedon* | 35.04 | 36.38 |
| Arizona | Mountain Chickadee | *Poecile gambeli* | 35.14 | 36.06 |
| Arizona | Red-faced Warbler | *Cardellina rubifrons* | 35.08 | 36.2 |
| Arizona | Western Bluebird | *Sialia mexicana* | 35.42 | 36.43 |

Table S2. Summary output of individual tropical (blue) and north temperate (red) species tested for differences between treatment and control nests in egg mass loss (g) and mass-specific metabolic rate (mL $O_2$ $h^{-1}$) using ANOVA with a random factor of year. Significant and marginally significant differences are denoted with (*) and (`) respectively

| **Species** | **Dependent variable** | ***F* value** | ***P* value** |
|---|---|---|---|
| Bornean Stubtail | Egg mass | 0.612 | 0.515 |
| | Mass-specific metabolic rate | 0.910 | 0.440 |
| | | | |
| Mountain Wren-babbler | Egg mass | 2.393 | 0.261 |
| | Mass-specific metabolic rate | 4.26 | 0.090` |
| | | | |
| Chestnut-crested Yuhina | Egg mass | 0.0005 | 0.983 |
| | Mass-specific metabolic rate | 0.676 | 0.471 |
| | | | |
| Cordilleran Flycatcher | Egg mass | 0.730 | 0.440 |
| | Mass-specific metabolic rate | 9.102 | 0.037* |
| | | | |
| Grey-headed Junco | Egg mass | 0.520 | 0.545 |
| | Mass-specific metabolic rate | 0.929 | 0.407 |
| | | | |
| House Wren | Egg mass | 7.458 | 0.034* |
| | Mass-specific metabolic rate | 1.016 | 0.352 |
| | | | |
| Mountain Chickadee | Egg mass | 0.023 | 0.888 |
| | Mass-specific metabolic rate | 79.65 | 0.012 * |
| | | | |
| Red-faced Warbler | Egg mass | 4.666 | 0.083` |
| | Mass-specific metabolic rate | 0.362 | 0.568 |
| | | | |
| Western Bluebird | Egg mass | 6.340 | 0.032* |
| | Mass-specific metabolic rate | 1.444 | 0.26 |

Table S3. Common and scientific names for 9 bird species breeding at our tropical (Malaysia) and a north temperate (Arizona) field sites. Total number of paired treatment and control nests (*n*) are shown for our heating and swap experiments.

| **Site** | **Common Name** | **Scientific Name** | **Heating Experiment *n*** | **Swap Experiment *n*** |
|---|---|---|---|---|
| Malaysia | Bornean Stubtail | *Urosphena whiteadi* | 3 | 4 |
| Malaysia | Mountain Wren-babbler | *Napothera crassa* | 3 | --- |
| Malaysia | Chestnut-crested Yuhina | *Yuhina everetti* | 4 | 4 |
| Arizona | Cordilleran Flycatcher | *Empidonax occidentalis* | 5 | --- |
| Arizona | Grey-headed Junco | *Junco hyemalis* | 4 | --- |
| Arizona | House Wren | *Troglodytes aedon* | 5 | --- |
| Arizona | Mountain Chickadee | *Poecile gambeli* | 3 | --- |
| Arizona | Red-faced Warbler | *Cardellina rubifrons* | 7 | --- |
| Arizona | Western Bluebird | *Sialia mexicana* | 8 | --- |
| | Totals | | 42 | 8 |

Table S4. Comparison of fit between linear mixed models testing the effect of incubation period differences on embryonic period changes between treatment and control nests for nine species of breeding songbirds at two latitudes (see methods). Models were tested (a) with intercept values free to vary and (b) forcing all intercepts through zero.

| | Model with free varying intercepts | Model with intercepts forced through 0 |
|---|---|---|
| **AIC** | 154.61 | 153.67 |
| **BIC** | 161.57 | 158.88 |
| **$r^2$** | 0.91 | 0.88 |
| **SE** | 0.23 | 0.16 |
| **F-statistic** | 20.10 | 60.75 |

Figure S1. Picture of the set up needed for the heating experiment here uncovered from camouflaging materials for showing purposes. The heating device (solid red arrow) wraps around the nest cup where a fake egg connected to a probe (red star) records temperature experienced by the embryos during incubation. A thermostat connected to the circuit (dashed red arrow) regulates the energy input from the battery to the heating device.  Notice the iron mesh surrounding the nest to reduce sample loss due to predation.

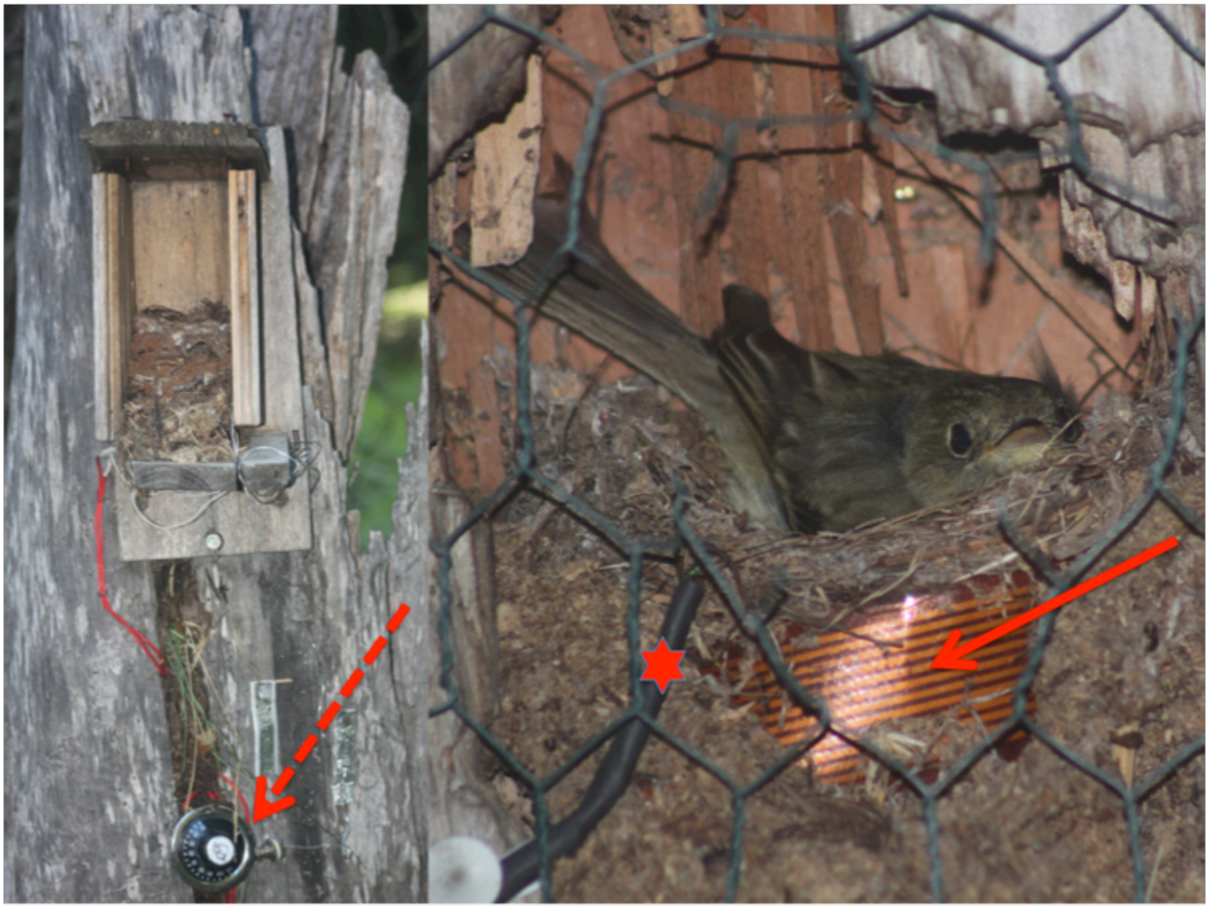

Figure S2. Phylogenetic relationships among north temperate (red) and tropical (blue) bird species used in the present study. The majority rule consensus tree was computed in program Mesquite using 1,000 trees obtained from BirdTree.org.

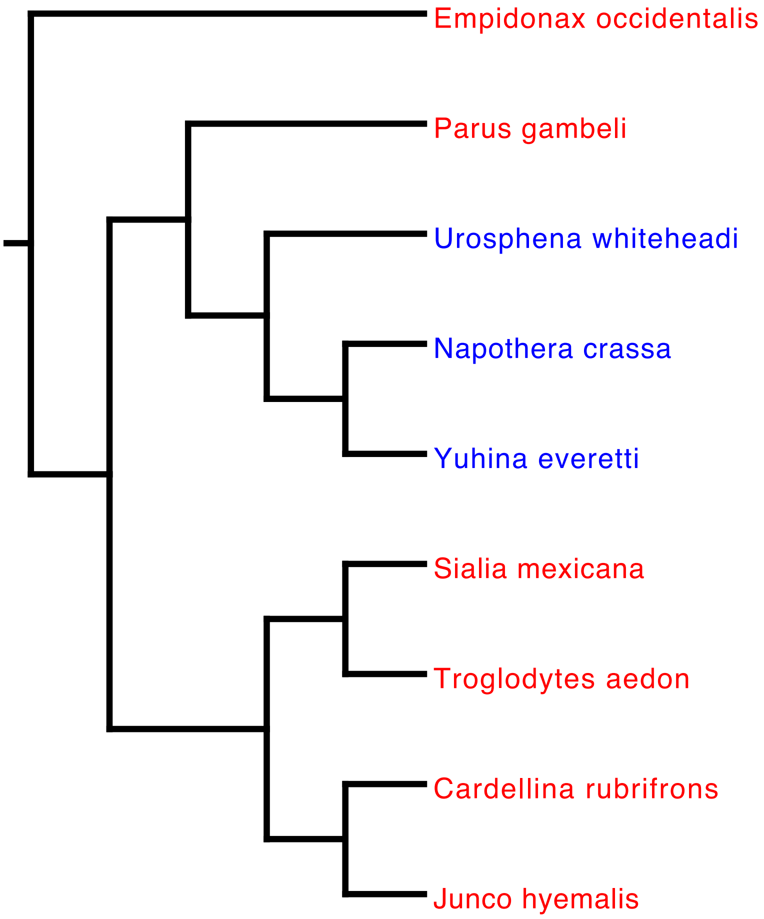

Supplement: Supplementary file 1 — Supplementary material [file 41598_2017_885_MOESM1_ESM.pdf]
